# ASSOCIATIONS OF OBJECTIVE MULTISENSORY IMPAIRMENT WITH COGNITIVE IMPAIRMENT: THE ARIC-NCS STUDY

**DOI:** 10.1093/geroni/igad104.0631

**Published:** 2023-12-21

**Authors:** Jason Smith, Vidyulata Kamath, Honglei Chen, Srishti Shrestha, Alison Abraham, Alden Gross, Nicholas Reed, Jennifer Deal

**Affiliations:** Johns Hopkins Bloomberg School of Public Health, Baltimore, Maryland, United States; Johns Hopkins University School of Medicine, Baltimore, Maryland, United States; College of Human Medicine, Michigan State University, East Lansing, Michigan, United States; University of Mississippi Medical Center, Jackson, Mississippi, United States; Colorado School of Public Health, Aurora, Colorado, United States; Johns Hopkins University Bloomberg School of Public Health, Baltimore, Maryland, United States; Johns Hopkins Bloomberg School of Public Health, Baltimore, Maryland, United States; Johns Hopkins University, Baltimore, Maryland, United States

## Abstract

Sensory impairments frequently co-occur among older adults. Characterizing the combined impact of a multisensory phenotype on cognitive impairment could help elucidate a potential mode of pathogenesis. We investigated associations between multisensory impairment (co-occurring, objectively-measured hearing loss [pure-tone average ≥25 dB], vision loss [presenting near or distance acuity >0.3 logMAR], anosmia [Sniffin’ Sticks test score ≤6], and peripheral nerve damage [≥1 insensate site on left or right foot]) and cognitive impairment (mild cognitive impairment [MCI] or dementia). Our study sample consisted of Black and White adults aged 70+ years in the Atherosclerosis Risk in Communities (ARIC) study; a longitudinal, community-based cohort. We estimated prevalence ratios (PRs) of baseline (ARIC visit 6, 2016-2017) expert-adjudicated MCI/dementia and odds ratios (ORs) of progression to MCI/dementia through ARIC visit 7 (2019) by number of impairments (0 [reference], 1, 2, 3 or more) using Poisson and logistic regression models, respectively. Overall, among 814 participants (age range: 71-93 years, 62% female, 41% Black race), an increasing number of impairments was associated with increased prevalence of baseline MCI/dementia (1 impairment, PR=1.14 [95% CI: 0.67-2.01]; 2 impairments, PR=1.53 [95% CI: 0.87-2.79]; ≥3 impairments, PR=2.26 [95% CI: 1.18-4.38]; p-trend: .007) and increased progression to MCI/dementia (1 impairment, OR=1.47 [95% CI: 0.67-3.49]; 2 impairments, OR=1.64 [95% CI: 0.69-4.12]; ≥3 impairments, OR=5.68 [95% CI: 2.18-15.52]; p-trend: .001). These findings suggest multiple concurrent sensory impairments contribute additively to greater MCI/dementia prevalence, signaling the importance of considering multiple impairments together in studies of the contribution of sensory losses to cognitive impairment.

